# Can genomic signatures guide the selection of host‐specific agents for weed biological control?

**DOI:** 10.1111/eva.13760

**Published:** 2024-07-17

**Authors:** Nagalingam Kumaran, S. Raghu

**Affiliations:** ^1^ Commonwealth Scientific and Industrial Research Organization (CSIRO) Health and Biosecurity Brisbane Queensland Australia

**Keywords:** biocontrol, biosecurity, host range, invasive species, natural enemies, weed control

## Abstract

Biological control of weeds involves deliberate introduction of host‐specific natural enemies into invaded range to reduce the negative impacts of invasive species. Assessing the specificity is a crucial step, as introduction of generalist natural enemies into a new territory may pose risks to the recipient communities. A mechanistic understanding of host use can provide valuable insights for the selection of specialist natural enemies, bolster confidence in non‐target risk assessment and potentially accelerate the host specificity testing process in biological control. We conducted a comprehensive analysis of studies on the genomics of host specialization with a view to examine if genomic signatures can help predict host specificity in insects. Focusing on phytophagous Lepidoptera, Coleoptera and Diptera, we compared chemosensory receptors and enzymes between “specialist” (insects with narrow host range) and “generalist” (insects with wide host range) insects. The availability of genomic data for biological control agents (natural enemies of weeds) is limited thus our analyses utilized data from pest insects and model organisms for which genomic data are available. Our findings revealed that specialists generally exhibit a lower number of chemosensory receptors and enzymes compared with their generalist counterparts. This pattern was more prominent in Coleoptera and Diptera relative to Lepidoptera. This information can be used to reject agents with large gene repertoires to potentially accelerate the risk assessment process. Similarly, confirming smaller gene repertoires in specialists could further strengthen the risk evaluation. Despite the distinctive signatures between specialists and generalists, challenges such as finite genomic data for biological control agents, ad hoc comparisons, and fewer comparative studies among congeners limit our ability to use genomic signatures to predict host specificity. A few studies have empirically compared phylogenetically closely related species, enhancing the resolution and the predictive power of genomics signatures thus suggesting the need for more targeted studies comparing congeneric specialists and generalists.

## 1 INTRODUCTION

Natural enemies play a key role in regulating populations and maintaining ecosystem functions. However, invasive species that are free from their natural predators in novel environments can disrupt ecosystems by outcompeting native species and establishing dominance (Weidlich et al., 2020). Classical biological control aims to mitigate this disruption by deliberately introducing natural enemies from the native range of invaders into their invaded range. For example, in weed biological control, insects or plant pathogens associated with host plants in their native range are introduced into the invaded range to increase the natural enemy assemblage (Müller‐Schärer & Schaffner, 2008). These natural enemies without their antagonists become potential biological control agents and contribute to reducing the negative impacts of invasive weeds on ecosystems at a landscape scale (Clewley et al., 2012; McFadyen, 1998; Schaffner et al., 2020; Schwarzländer et al., 2018).

Classical weed biological control programs use insects (referred as “biological control agents” or “agents”) that are extreme host specialists (i.e., feed and develop primarily on the target weed). While a narrow host range is common in phytophagous insects, some species are generalists, feeding on many plants from multiple genera or families, as with many pests (Jaenike, 1990; Kennedy & Storer, 2000). Consequently, potential biological control agents are subjected to host specificity testing before being introduced into the invaded range of weeds to avoid unintended non‐target risks to the recipient communities (e.g., native plant species). While current host‐specificity testing processes correctly predicts host range of biological control agents (Fowler et al., 2004; Hinz et al., 2019; Paynter et al., 2004, 2018; Sheppard et al., 2005; Wheeler & Madeira, 2017), ongoing advances in science enable us to continue to adaptively refine our characterization of risks and benefits in the use of specialist insect herbivores in classical weed biological control (Fowler et al., 2012).

**FIGURE 1:**
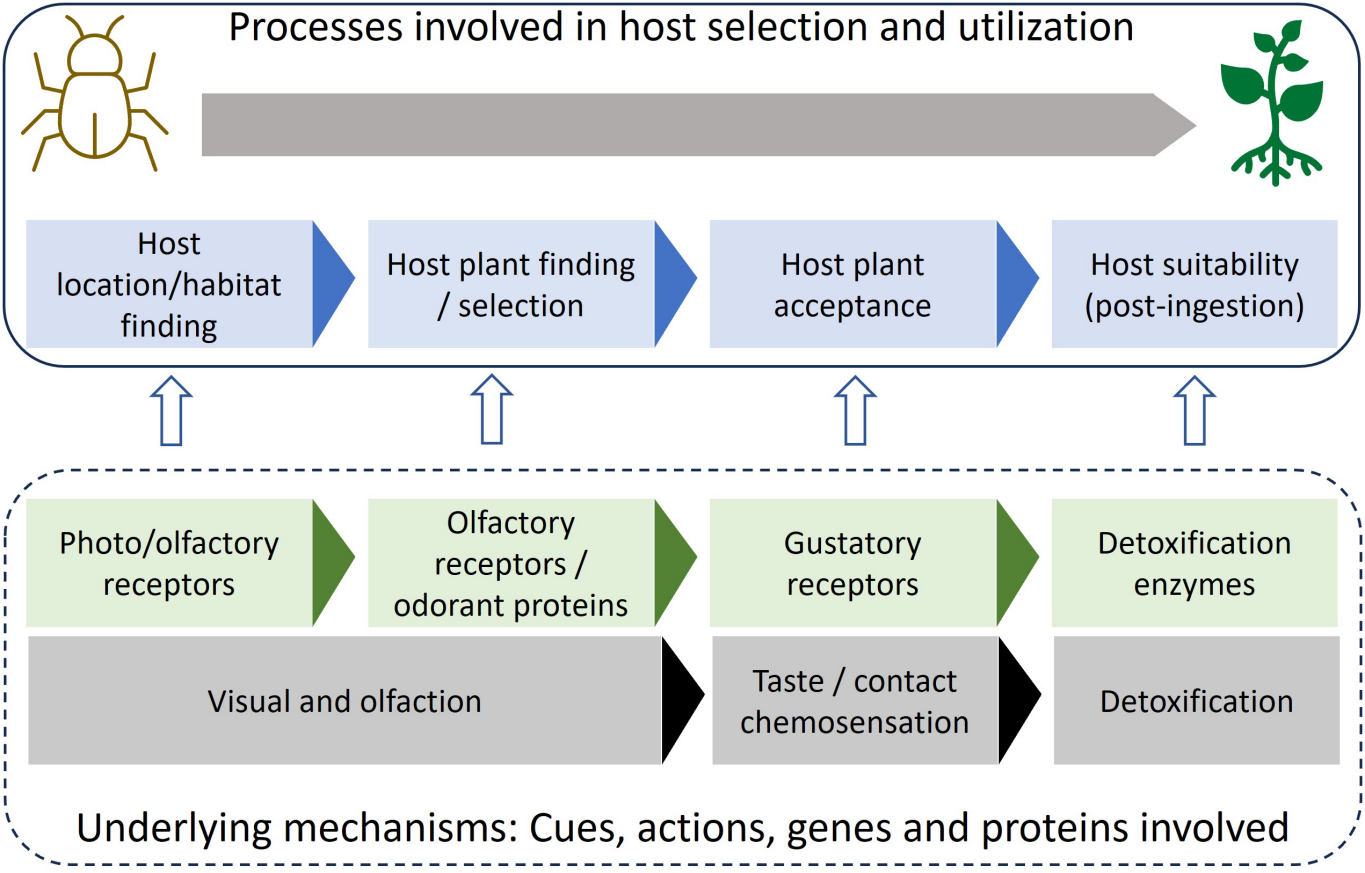
Conceptual representation of the catenary host use process in herbivorous insects, and the receptors and proteins involved in each step of the process.

Developing a mechanistic understanding of the catenary host use process can provide insights into predicting host specificity of biological control agents bolstering confidence in risk assessment. The key steps for successful hosts use are finding the habitat the potential host is in, finding the potential host, accepting the host for feeding or oviposition, and for immature stages, suitability of the potential host for the completion of development (Kennedy, 1965; Schoonhoven et al., 2005; Thorsteinson, 1960), and each of these steps involves perceiving complex sensory cues (Figure 1) (Bernays & Chapman, 1994; Visser, 1988). While insects use visual and olfactory cues to identify habitats and host plants (Webster & Cardé, 2017), host acceptance and host suitability are dependent on the ability of insects to perceive gustatory stimuli and to detoxify host plant toxins (Zwölfer & Harris, 1971).

Perceiving cues from host plants is predominantly mediated by chemosensory receptors and enzymes, which are a physiological mechanism underpinning the host use process (Leal, 2013; Prokopy & Owens, 1983). Both historical and contemporary studies investigating host use from a mechanistic perspective have found that these receptors and enzymes vary in quantity between insects with narrow and wide host ranges. Historical predictions suggest that several primary metabolites induce feeding in generalist insects (Chapman, 1982; Dethier, 1948; Futuyma & Peterson, 1985; Rees, 1969), and contemporary studies have highlighted that generalists have expanded chemosensory gene families as they utilize a diversity of host plants and have developed diverse mechanisms to circumvent plant defenses (Birnbaum & Abbot, 2020; Cheng et al., 2017; Gouin et al., 2017; Simon et al., 2015). In contrast, specialists seem to maintain more targeted mechanisms for processing specific plant stimuli or toxins (Govind et al., 2010; Heidel‐Fischer et al., 2019; Li et al., 2004; Vogel et al., 2014). Insights from these evolutionary studies could serve as a basis for potentially selecting host‐specific agents for weed biological control.

Our aim in this paper was to systematically compare the size and functional difference of gene repertoires between specialists and generalists and search for genomics signatures of host use. We postulate that generalists harbor a greater number of receptors and enzymes. In contrast, specialists use fewer receptors and proteins to perceive cues within their strict host range. Understanding this difference is important not only to bolster confidence in predicting the host specificity of agents, but also because it could accelerate the screening of candidate biological control agents. We used the term “specialists” or “generalists” to refer to insects with narrow and wide host ranges, respectively, consistent with their usage in the studies reviewed here. Consequences of using these terms in their broad sense in the context of weed biological control, along with potential challenges of using genomic signatures as predictors of host specificity, are discussed.

## 2 LITERATURE REVIEW, DATA SORTING, AND DATA ANALYSIS

We reviewed literature published until 2023 through Scopus using search terms [“chemosensory proteins” or “olfactory receptors” or “gustatory receptors” or “odorant binding proteins” or “cytochrome p450”] AND [“host breadth” or “specialist” or “generalist” or “host specialization” or “monophagous” or “oligophagous” or “polyphagous”] AND [“phytophagous” or “herbivorous”] AND [“Coleoptera” or “Diptera” or “Lepidoptera”]. Due to the extensive transcriptome information available for insects, we limited our investigation to species within the Lepidoptera, Coleoptera, and Diptera, which collectively represent the main insect orders deployed for biological control of weeds (Schwarzländer et al., 2018). Despite using restrictive keywords, our search yielded 1261 records, from which we selected studies reporting counts of chemosensory proteins and detoxification enzymes.

After screening, we identified 55 relevant studies on Lepidoptera, 36 on Coleoptera, and 27 on Diptera for comparative analyses; these studies included independent empirical studies focused on specialists or generalists, empirical studies comparing specialist and generalist insects and review articles discussing difference in number of genes between specialists and generalists. We extracted data on number of olfactory receptors (ORs), odorant‐binding proteins (OBPs), gustatory receptors (GRs), and cytochrome P450s, the main physiological drivers mediating host use (Ali & Agrawal, 2012; Birnbaum & Abbot, 2020), to make comparisons across monophagous, oligophagous, and polyphagous species within each insect order. The terminologies used to define degree of specialization vary in the literature with “monophagous” and “oligophagous” categorized as specialists (narrow host range) and “polyphagous” as generalists (wide host range) (e.g., Dethier, 1954; Jaenike, 1990, Almeida‐Neto et al., 2011). For our analyses, we categorized species as “monophagous” if insects feeding on 1 or more plant species within a single genus, as “oligophagous” if species is restricted to feeding on two or more genera in a family, and as “polyphagous” if a species feed on host plants from two or more plant families, following criteria described by Cates (1980).

We used ANOVA to analyze means of ORs, OBPs, GRs, and cytochrome P450s, acknowledging that this assumes independence of data, which is likely violated given the phylogenetic relatedness of species within each insect order particularly when comparing congeners. We considered phylogenetically independent contrasts (PICs), but their application was hindered by the absence of nuclear loci and poor data resolution with mitochondrial loci for certain species. ANOVA at the order level was deemed a pragmatic compromise. Prior studies suggested that evolution of enzymes and receptors is independent of ancestral relationships between species (Ribeiro et al., 2023), and phylogenetic comparisons of families within the order indicated sufficient inter‐family differences (Rota et al., 2022; Wiegmann et al., 2011). However, we acknowledge that there may be variations among taxa and employing PICs could enhance analytical clarity when genomic data becomes available to take evolutionary relationships into account. This caveat is further considered under “challenges” later in the Discussion.

In addition to comparisons within Lepidoptera, Coleoptera, and Diptera, we compared differences in gene repertoires between congeners and between biocontrol agents and generalists. Genomic data are generally lacking for biological control agents (species that are known specialists and already released for weed control). However, transcriptome data were reported for the crofton weed fly *Procecidochares utilis* (Diptera) and the Alligator weed flea beetle *Agasicles hygrophila* (Coleoptera) (Gao et al., 2014; Jia et al., 2018). We obtained these data directly from authors and manually screened counts of ORs, GRs, OBPs, and P450s and compared that with data from oligophagous and polyphagous species within the same insect orders. We present our comparative analyses as descriptive case studies below.

## 3 CASE STUDY 1. COMPARISON OF GENERALISTS VERSUS SPECIALISTS WITHIN LEPIDOPTERA

Species in Lepidoptera have been extensively investigated for comparisons between specialists and generalists. Previous reviews on Lepidoptera have yielded mixed results on the hypothesis that generalists possess a greater number of receptors and enzymes than specialists. Investigating generalist *Helicoverpa armigera*, Xu et al. (2016) reported 180 GRs compared to 51 in specialist *Bombyx mori*, and 57, 49, and 40 in oligophagous *Heliconius melpomene*, *Plutella xylostella*, and *Danaus plexippus*, respectively.

Similar observations were made in other generalists. In *Spodoptera frugiperda*, the number of GRs and cytochrome P450s was relatively higher in comparison with other specialists (*B. mori*, *M. sexta*, *H. Melpomene*, and *D. plexippus*) (Gouin et al., 2017). Similarly, a greater number of GRs and cytochrome P450s were reported for the generalists *Helicoverpa zea* and *Spodoptera litura* in comparison to specialist *B. mori* (Cheng et al., 2017; Pearce et al., 2017). Conversely, though they found that the generalist *Operophtera brumata* appeared to have more cytochrome P450s than *B. mori*, Calla et al. (2017) reported an equal number of cytochrome P450s in generalist *Amyelois transitella* and specialist *Depressari pastinacella*.

**FIGURE 2:**
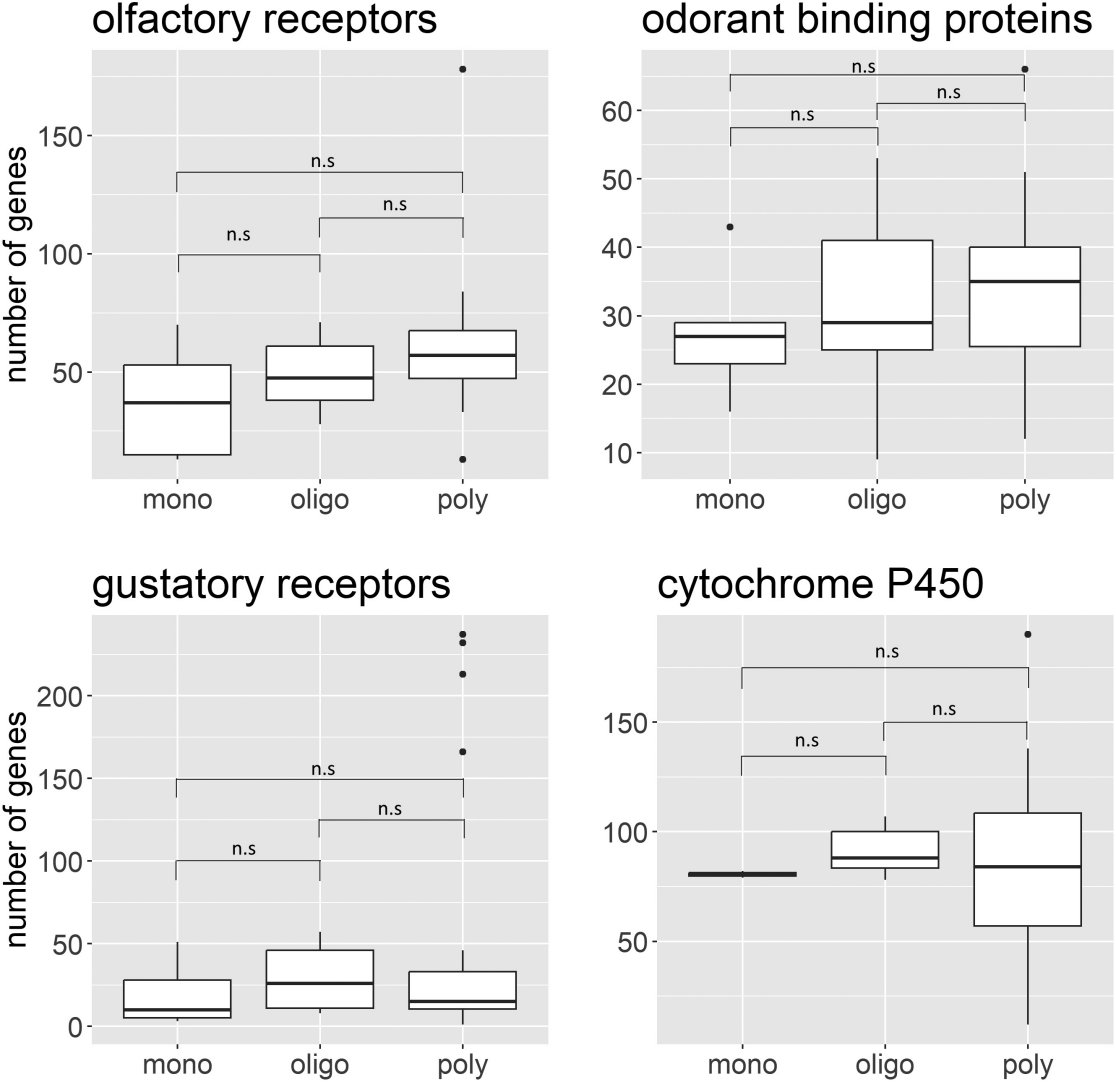
Number of olfactory receptors (*n* = 51), gustatory receptors (*n* = 33), odorant binding proteins (*n* = 42), and cytochrome P450s (*n* = 23) in monophagous (mono), oligophagous (oligo), and polyphagous (poly) Lepidopteran species. The box plot comprises the median line, interquartile range from 25th to 75th percentile (the bounding box), the minimum (25th percentile −1.5*interquartile range), and maximum whiskers (75th percentile +1.5*interquartile range) and outliers (the circles beyond the whiskers).

Upon scrutiny of previous reviews and comparison of 55 Lepidopteran species, we found limited evidence supporting the hypothesis that generalists possess greater numbers of ORs, GRs, OBPs, and cytochrome P450s, and this difference was not statistically different when the data were analyzed using Kruskal‐Wallis one‐way ANOVA (OR: H_2,50_ = 3.702, *p* = 0.157; GR: H_2,32_ = 0.723, *p* = 0.696; OBP: H_2,41_ = 0.095; *p* = 0.953; Cytochrome P450: H_2,22_ = 2.808; *p* = 0.246) (Figure 2). This is likely a result of ad hoc comparison of phylogenetically unrelated species and experimental artifacts. For example, the choice of tissues for RNA extraction varied between studies, such as larvae for *D. pastinacella* and only midgut RNA for the generalist *A. transitella* in the study by Calla et al. (2017).

## 4 CASE STUDY 2. COMPARISON OF GENERALISTS VERSUS SPECIALISTS WITHIN COLEOPTERA

Unlike Lepidoptera, studies comparing Coleoptera are limited. Comparing bark beetles, Andersson et al. (2019) reported fewer ORs, GRs, and OBPs in the specialist *Dendroctonus ponderosae* and *Agrilus planipennis*, relative to the generalist *Anoplophora glabripennis*. In another comparison involving a wide range of families within Coleoptera, Mitchell et al. (2020) demonstrated that the diversity of ORs is correlated with host breadth with fewer ORs in specialists when compared to generalists.

**FIGURE 3:**
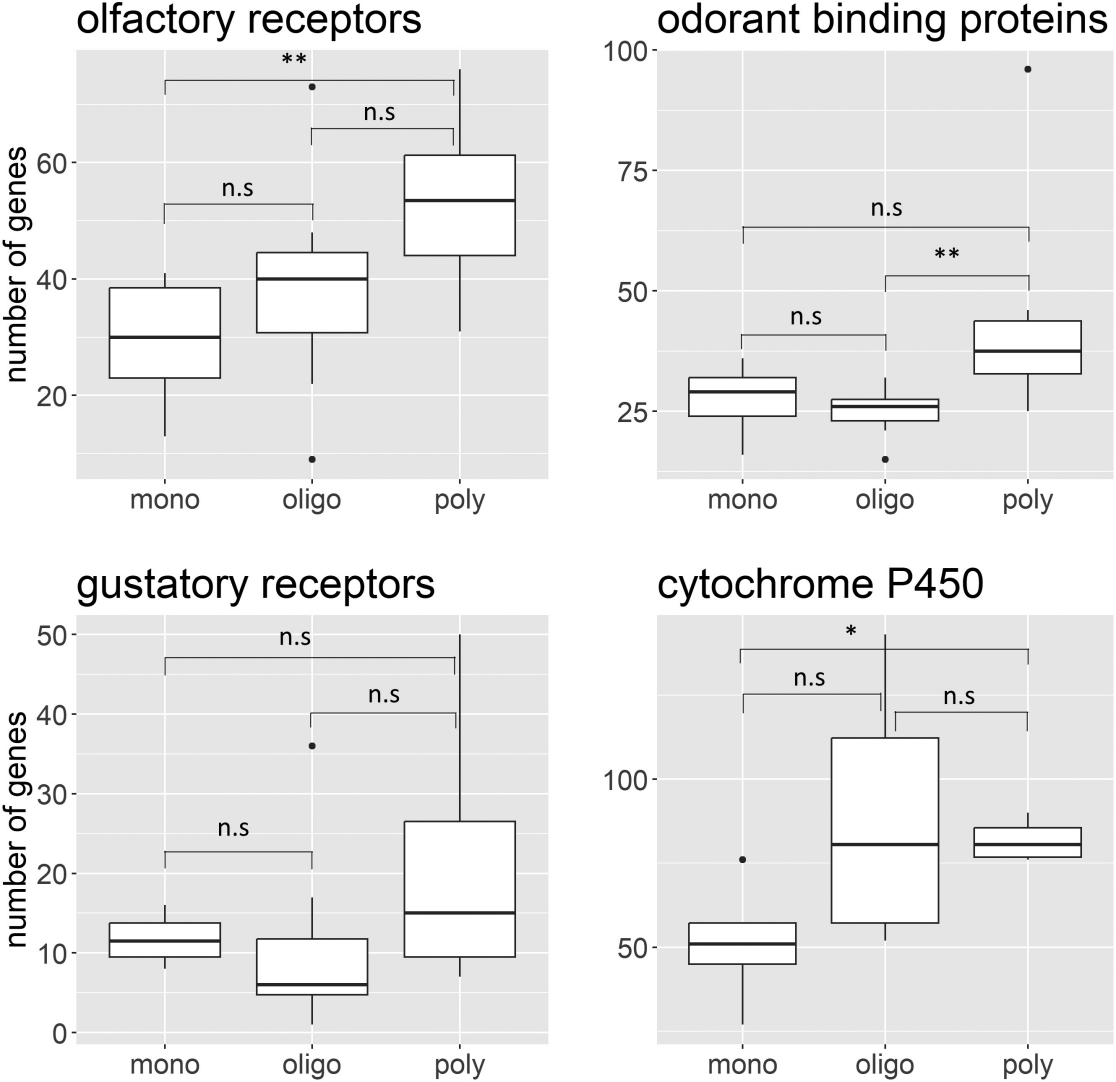
Number of olfactory receptors (*n* = 26), gustatory receptors (*n* = 19), odorant binding proteins (*n* = 27) and cytochrome P450s (*n* = 13) in monophagous (mono), oligophagous (oligo) and polyphagous (poly) Coleoptera species. The box plot comprises the median line, interquartile range from 25th to 75th percentile (the bounding box), the minimum (25th percentile −1.5*interquartile range), and maximum whiskers (75th percentile +1.5*interquartile range) and outliers (the circles beyond the whiskers).

We compared a total of 36 species with varied host ranges. For this synthesis, we only focused on phytophagous species and therefore a few insect groups were excluded from the analysis (e.g., stored grain pests). The results showed convincing evidence for specialists possessing fewer ORs and OBPs in Coleoptera (OR: H_2,25_ = 9.635, *p* = 0.008; OBP: H_2,26_ = 9.217; *p* = 0.009) (Figure 3). However, number of GRs were not significantly different (H_2,18_ = 4.099, *p* = 0.129) while the cytochrome P450s indicated a marginal increasing trend from specialists to generalists (H_2,12_ = 7.937; *p* = 0.012) (Figure 3).

## 5 CASE STUDY 3. COMPARISON OF GENERALISTS VERSUS SPECIALISTS WITHIN DIPTERA

While *Drosophila* species have been used as model systems in studies focused on host breadth, comparative analyses in phytophagous Diptera were rare in the published literature. Recently, Shi et al. (2022) analyzed host plant expansion in the Tephritidae and found a greater number of ORs, GRs and OBPs in generalist *Bactrocera dorsalis* and *Ceratitis capitata* when compared to the specialists *B. oleae*, *B. minax, Procecidochares utilis, Carpomya vesuviana* and *Zeugodacus cucurbitae*. Shi et al. (2022) also reported a greater number of cytochrome P450s in the polyphagous *B. dorsalis* and *C. capitata* than in the monophagous *B. oleae*.

**FIGURE 4:**
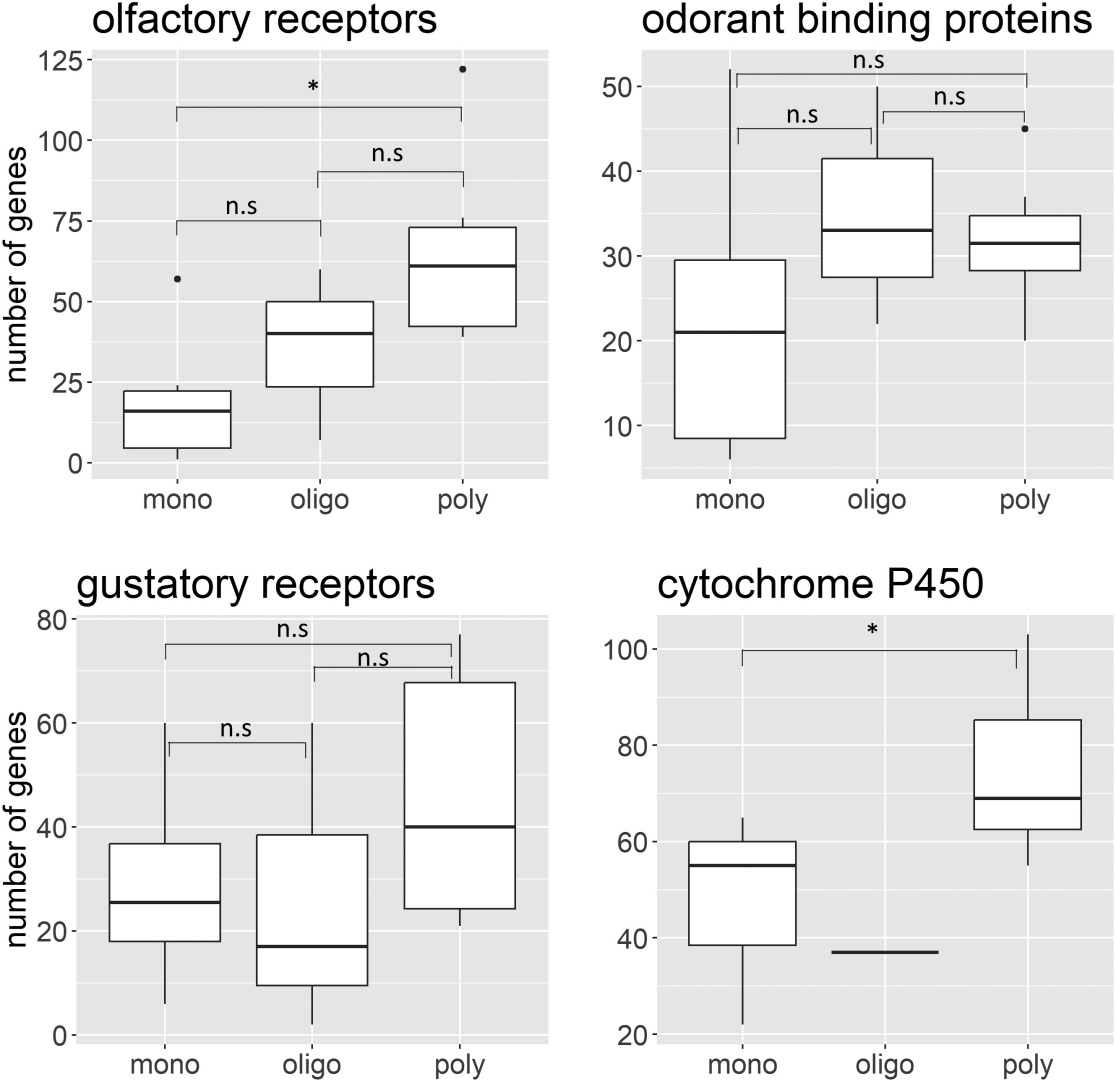
Number of olfactory receptors (*n* = 19), gustatory receptors (*n* = 13), odorant binding proteins (*n* = 25), and cytochrome P450s (*n* = 10) in monophagous (mono), oligophagous (oligo), and polyphagous (poly) Dipteran species. The box plot comprises the median line, interquartile range from 25th to 75th percentile (the bounding box), the minimum (25th percentile −1.5*interquartile range), and maximum whiskers (75th percentile +1.5*interquartile range) and outliers (the circles beyond the whiskers).

We analyzed data from 27 species within Diptera. Similar to Lepidoptera and Coleoptera, non‐phytophagous species (e.g., *Drosophila melanogaster*) were excluded from the analysis. We found fewer ORs in specialists when compared to generalists (H_2,18_ = 8.876, *p* = 0.012) (Figure 4). This aligns with the results of Shi et al. (2022) and a recent study that showed increased ORs in generalist *B. dorsalis* when compared to specialist *B. minax* (Wang et al., 2022). However, the number of GRs and OBPs were not significantly different (GR: H_2,12_ = 0.485, *p* = 0.785; OBP: H_2,24_ = 2.563; *p* = 0.277) (Figure 4). Cytochrome P450 data were available for only one oligophagous species, thus we performed Welch *t*‐test to compare the means of monophagous and polyphagous species. The data showed a significant difference in the number of cytochrome P450s between specialists and generalists (*t* = −2.452, *p* = 0.047).

## 6 CASE STUDY 4. COMPARING GENERALIST AND SPECIALIST CONGENERS

**FIGURE 5:**
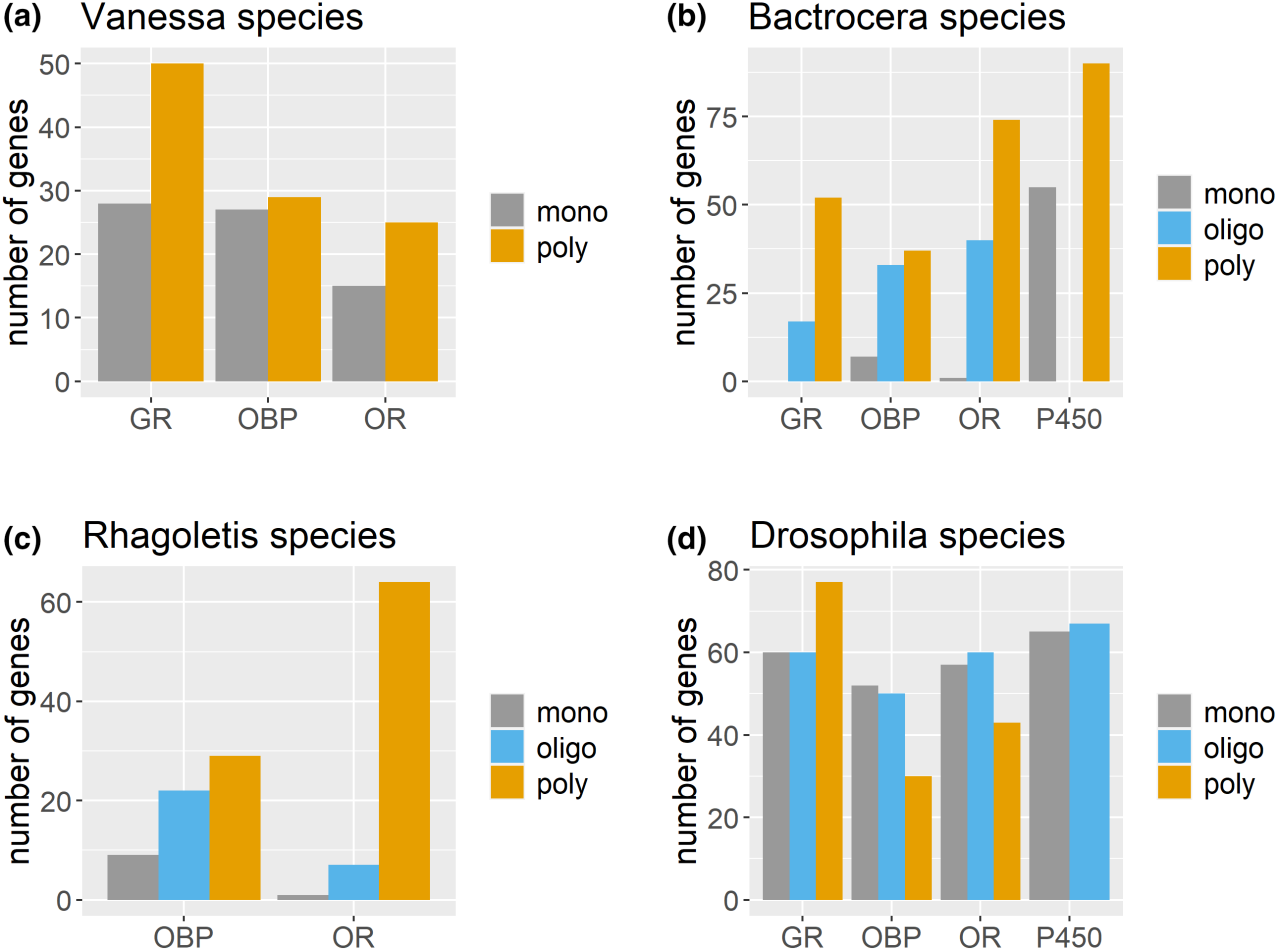
Difference in number of gene repertoires between generalist and specialist congeners; (a) *Vanessa indica* (monophagous) versus *V. cardui* (polyphagous); (b) *Bactrocera oleae* (monophagous) versus *B. minax* (oligophagous) and *B. dorsalis* (polyphagous); (c) *Rhagoletis suavis* (monphagous) versus *R. pomonella* (oligophagous) vs *R. zephyria* (polyphagous); (d) *Drosophila sechellia* (monophagous) versus *D. elegans* (oligophagous) versus *D. suzukii* (polyphagous) (data adapted from original studies referenced in Table S4) (GR, gustatory receptors; mono, monophagous; OBP, odorant binding proteins; oligo, oligophagous; OR, odorant receptors; P450, cytochrome P450s; poly, polyphagous).

Congeners may offer more reliable data for the comparison of specialists and generalists than distantly related species (Ali & Agrawal, 2012). In a study by Suzuki et al. (2018), gene repertoires of the generalist *Vanessa cardui* were compared with those of its specialist congener *V. indica*, revealing a greater number of ORs and GRs in the generalist species (Figure 5a). In another congeneric comparison, Zhang et al. (2015) compared the specialist *Helicoverpa assulta* with its generalist congener *H. armigera*. Although ORs and OBPs did not differ in this pair, a separate study documented 18 GRs in *H. assulta* (Xu et al., 2015) compared to 180 in *H. armigera* (Xu et al., 2016).

We conducted comparisons of specialist and generalist species within the genera *Bactrocera*, *Rhagoletis*, and *Drosophila*. The disparities in gene repertoires were particularly pronounced in *Bactrocera* when a monophagous species (*B. oleae*) was compared with the oligophagous (*B. minax*) and polyphagous (*B. dorsalis*) congeners (Figure 5b). While data for GRs and cytochrome P450s were unavailable for *Rhagoletis* species, the number of ORs and OBPs were lower in monophagous species (*R. suavis*) than in oligophagous (*R. pomonella*) and polyphagous (*R. zephyria*) species (Figure 5c). In contrast, distinctions between specialist (*D. sechellia*) and generalist *Drosophila* species (*D. erecta* and *D. suzukii*) were less apparent (Figure 5d).

The data from *Bactrocera*, *Vanessa*, and *Rhagoletis* provided compelling evidence that genomic signatures vary based on the host range. Although the number of cytochrome P450s in *B. oleae* is lower compared to its generalist congener *B. dorsalis*, we expected that *B. oleae* harbor a greater number of detoxification genes to overcome resistance from its *Olea* hosts, known for their high phenolics and phytotoxins content (Noce et al., 2012). The detoxification transcripts of *B. oleae* perhaps constitute a cluster of enzymes specifically tailored to toxins in *Olea* spp., displaying a higher magnitude of action on certain compounds, akin to *Papilio polyxenes* discussed below in case study 5.

## 7 CASE STUDY 5. STRUCTURAL AND FUNCTIONAL DIFFERENCE IN RECEPTORS AND ENZYMES

**FIGURE 6:**
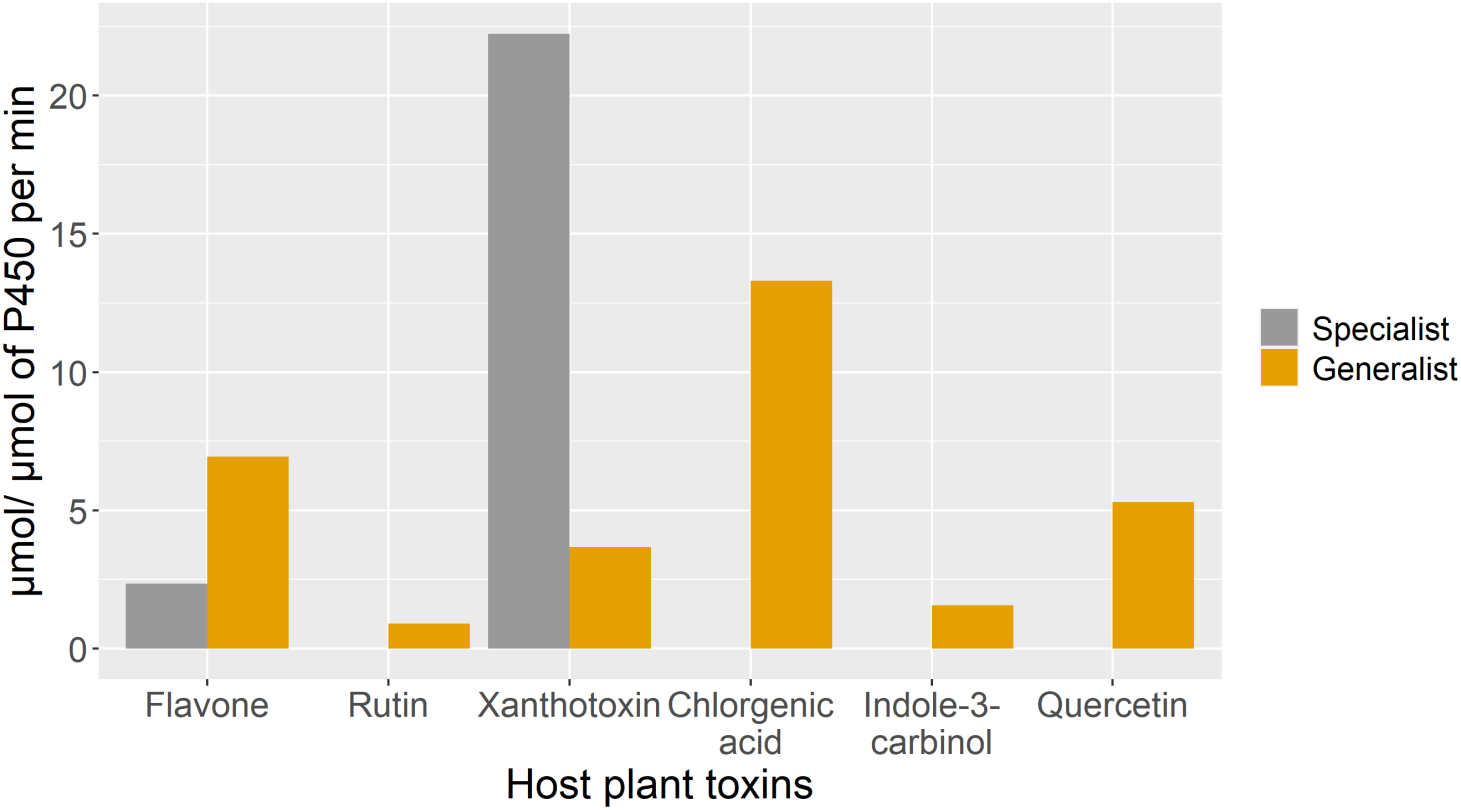
Difference in metabolic kinetics of detoxifying proteins of plant defense chemicals (CYP6B1 and CYP6B8) between specialist *P. polyxenes and* generalist *H. zea* (graphs reproduced using the data from Li et al., 2004).

In addition to quantitative differences, specialists and generalists exhibit structural and functional differences further supporting the view that host breadth is underpinned by mechanistic factors. For example, specialists rely on efficient and rapid detoxification and/or sequestration of toxins (Engler et al., 2000; Ratzka et al., 2002; Sasabe et al., 2004), whereas generalists employ generic chemical modification, degradation, and excretion tactics to detoxify a broad range of defense compounds (Badenes‐Perez et al., 2013; Dussourd, 1999; Francis et al., 2005). This contrast is exemplified in the case of the specialist *P. polyxenes* and the generalist *H. zea* (Li et al., 2004). The cytochrome P450 (CYP6B8) of *H. zea* metabolizes a suite of plant toxins (xanthotoxin, quercetin, flavone, chlorogenic acid, indole‐3‐carbinol, and rutin), whereas the cytochrome P450 (CYP6B1) of *P. polyxenes* metabolizes only xanthotoxin and flavone. The structure of CYP6B1 differs from that of CYP6B8, and the metabolic kinetics of CYP6B1 are also significantly greater than those of CYP6B8 (Figure 6).

Structural differences and an increased response to specific stimuli have been reported in other systems as well. The specialist parsnip webworm *D. pastinacella* metabolized a substantially greater amount of xanthotoxin compared with the generalist *Trichoplusia ni*, when identical concentrations of xanthotoxin were provided to both (Lampert et al., 2011). Similarly, the specialist *Drosophila erecta* displayed heightened sensitivity toward volatiles from its host *Pandanus* due to quantitative and structural differences in its olfactory sensory neurons (Linz et al., 2013).

## 8 CASE STUDY 6. COMPARING SPECIALIST WEED BIOLOGICAL CONTROL AGENTS WITH GENERALISTS

Transcriptome information for biological control agents is generally scarce, with notable exceptions being the crofton weed fly *P. utilis* and the Alligator weed flea beetle *A. hygrophila* (Gao et al., 2014; Jia et al., 2018). We compared the gene repertoires of these two species with generalist species using the data from Diptera for *P. utilis* and from Coleoptera for *A. hygrophila*. Mean data from oligophagous and polyphagous categories reviewed above under case studies were used for this comparison treating biological control agents as extreme specialists. These biocontrol agents were deemed specialists based on the results from non‐target screening that demonstrated host specificity toward target weeds.

**FIGURE 7:**
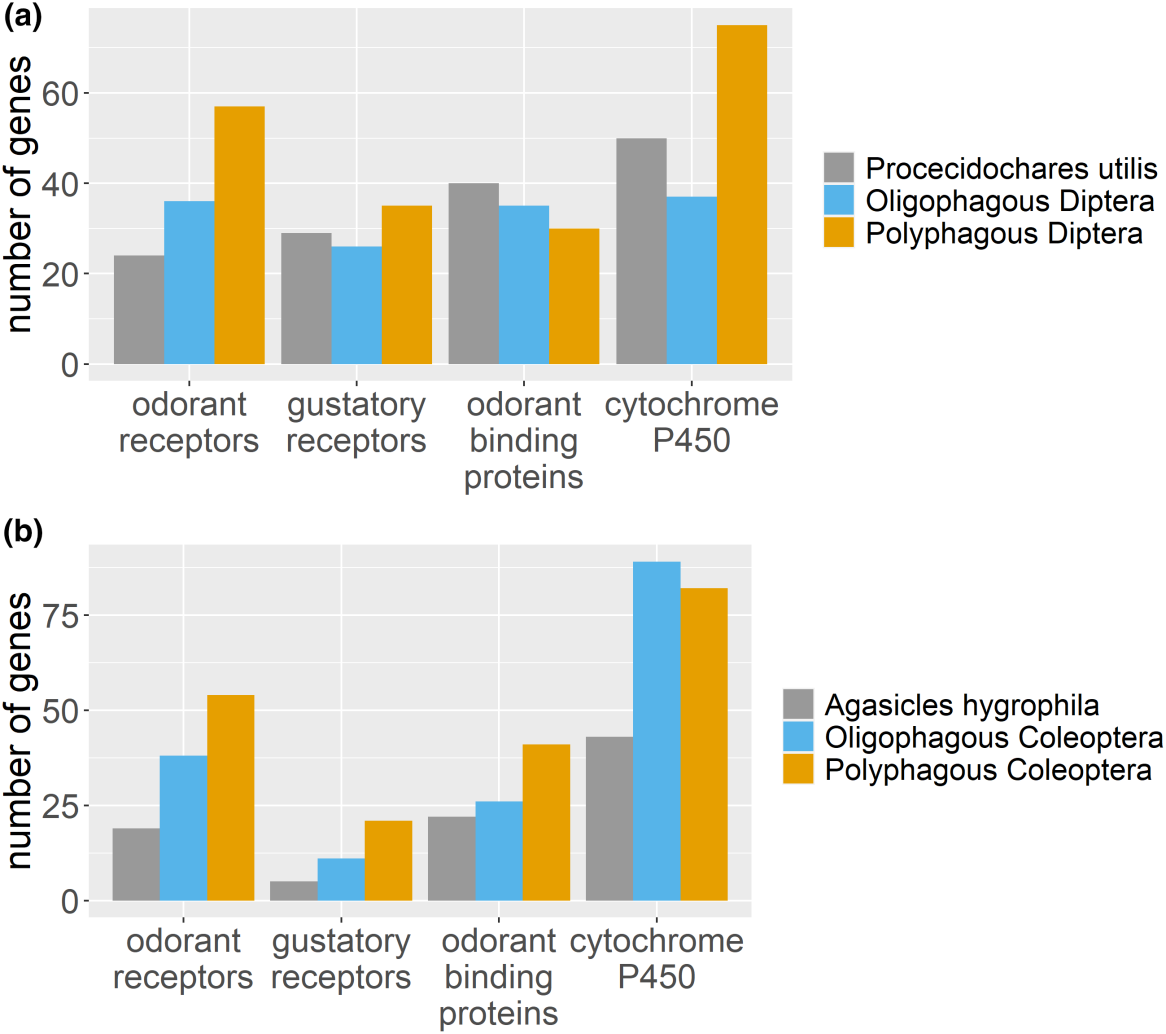
Biological control agents compared with generalist insects (a) number of genes in crofton weed gall fly, *Procecidochares utilis* in comparison with oligophagous and polyphagous Diptera (b) number of genes in Aligator weed flea bettle, *Agasicles hygrophila* in comparison with oligophagous and polyphagous Coleoptera.

The number of ORs and cytochrome P450s in *P. utilis* was lower than in polyphagous Diptera (Figure 7a). This disparity in ORs between specialists and generalists was more pronounced when *A. hygrophila* was compared with oligophagous and polyphagous species in Coleoptera (Figure 7b) providing further evidence for difference in gene repertoires between specialists and generalists.

## 9 CHALLENGES IN PREDICTING HOST SPECIFICITY OF BIOLOGICAL CONTROL AGENTS

Based on the trends noticed in the case studies, a greater number of receptors and enzymes seems to indicate a polyphagous habit, and this could be used to prioritize potential biological control agents. However, this interpretation in challenged by the anomalies discussed below, therefore a more nuanced exploration of the link between genomic signatures and host breadth is essential to confidently predict host specificity.

Comparing phylogenetically distant specialists and generalists that differ in various evolutionary aspects, in addition to their feeding biology, introduces inherent “phylogenetic noise” and makes the interpretations challenging (Ali & Agrawal, 2012). While comparing congeners can limit this noise, only a few studies have conducted such a pair‐wise empirical study using phylogenetically closely related specialists and generalists (e.g., Orsucci et al., 2018; Suzuki et al., 2018; Zhang et al., 2015). Other comparisons discussed in the case studies above relied on data from independent studies, employing different experimental designs, rearing methods, tissues, and sequencing approaches, potentially confounding the data. For instance, studies that have used adults or adult tissues (e.g., antennae) may have captured ORs and OBPs that adults use to locate host plants but may have overlooked GRs and cytochrome P450s that are rich in larval tissues. This ad hoc comparison underscores the need for developmental stage‐specific investigations, especially in species where adults and immature stages may rely on different host plants, or where only one life stage is herbivorous.

In addition to the methodological differences in the underlying molecular data, classifying certain species as specialists, despite using multiple host plants, raises ecological versus biological control distinctions. The black swallowtail butterfly (*P. polyxenes*), deemed an ecological specialist, is unlikely to meet the criteria for a biological control agent (Futuyma, 1976; Slansky Jr, 1976). This debate can be extended to biological control, where agents may be considered host‐specific enough to pose low/negligible risks in certain geographical locations due to the absence of closely related non‐target plant species, emphasizing the need for careful consideration when treating biological control agents as specialists.

Predicting host specificity is further complicated by the predominance of ecological drivers of host specialization relative to the intrinsic mechanisms governing host use. Theory predicts that host specialization is generally a species property controlled by the intrinsic factors (Jaenike, 1990; Mustaparta, 1992), and recent studies such as Orsucci et al. (2018), highlighting the presence of unique olfactory receptor genes mediating host discrimination in specialist *Ostrinia nubilalis* when compared with generalist *O*. *scapulalis*, affirm the dominant role of intrinsic factors. However, other views on the importance of ecological factors in shaping host specialization warrant consideration (Forister et al., 2012; Smiley, 1978).

## 10 FUTURE DIRECTIONS

Despite the caveats outlined above, the magnitude of structural and functional differences in gene repertoires based on the host ranges of phytophagous insects suggests that the abundance of chemosensory receptors and enzymes can be used to differentiate specialists from generalists. Future studies similar to Orsucci et al. (2018), Suzuki et al. (2018) and Zhang et al. (2015) could mitigate the experimental artefacts discussed above and controlling for “phylogenetic noise” may strengthen inference. More understanding can be gained by investigating the many cases where biological control agents can be compared with their congeneric generalists (e.g., *Spodoptera pectinicornis* vs. *S. litura*, *Paraponyx diminutalis* vs. *Paraponyx* spp., *Leptinotarsa defecta* or *L. texana* vs. *L. decemlineata*, *Liriomyza sonchi* vs *L. trifolii* and *Ophiomyia lantanae* or *O. camarae* vs. *O. phaseoli*). Targeted studies comparing specialist–generalist species pairs, where they are fed on same plant species to potentially avoid host‐induced variations, could provide rich insights on the use of this approach to predict host specificity in classical weed biological control.

## CONFLICT OF INTEREST STATEMENT

The authors declare that they have no known competing financial interests or personal relationships that could have appeared to influence the work reported in this paper.

## Data Availability

Review of already published data. Data compiled for this synthesis are available from authors upon reasonable request.
